# Integrin αVβ3 Function Influences Citalopram Immobility Behavior in the Tail Suspension Test

**DOI:** 10.3389/fnins.2019.00070

**Published:** 2019-02-06

**Authors:** Hope Pan, Michael R. Dohn, Rody Kingston, Ana M. D. Carneiro

**Affiliations:** Department of Pharmacology, Nashville, TN, United States

**Keywords:** antidepressant, TST, signaling network, integrin, citalopram, mouse model

## Abstract

Human studies first identified genetic and expression interactions between integrin β3 and serotonin (5-HT) transporter (SERT) genes. This association has been further strengthened by our discovery that integrin β3-containing receptors (αvβ3) physically interact with, and thereby define, a subpopulation of SERTs that may represent the main target of selective serotonin reuptake inhibitors (SSRIs). In this study, we examine how integrin αvβ3 function influences the behavioral response to the highly SSRI citalopram in the tail suspension test. Mice bearing a conditional deletion of the integrin β3 gene in neurons, or those expressing a constitutively active αvβ3 receptor, have decreased sensitivity to citalopram, when compared to wild-type littermates. To identify potential signaling pathways downstream of integrin αvβ3 that could be altered in these mouse lines, and consequently influence citalopram response *in vivo*, we performed antibody array analyses of midbrain synaptosomes isolated from mice bearing genetically altered integrin β3. We then pharmacologically targeted focal adhesion (FAK) and extracellular-signal-regulated (ERK) kinases and determined that FAK and ERK activity are critical for the actions of citalopram. Taken together, our studies have revealed a complex relationship between integrin αvβ3 function, SERT-dependent 5-HT uptake, and the effective dose of citalopram in the TST, thus implicating a role for integrin signaling pathways in the behavioral response to SSRIs.

## Introduction

Multiple genetic and environmental factors influence antidepressant response (Keers et al., 2011; Klengel and Binder, 2013). The tail suspension test (TST) is a simple and quick paradigm with strong predictive validity for the positive therapeutic outcomes for most antidepressants, including tricyclic and selective serotonin reuptake inhibitors (SSRIs) (Cryan et al., 2005; Castagne et al., 2011). With the exception of modifications in the serotonin system, there is no obvious genetic link between alterations in immobility time in the TST by acute antidepressant administration and reduction of symptoms upon chronic SSRI treatment.

We first identified the integrin β3 subunit as a modulator of peripheral and central serotonin homeostasis via its interactions with the high-affinity serotonin transporter (SERT) (Carneiro et al., 2008; Whyte et al., 2014; Mazalouskas et al., 2015; Dohn et al., 2017). Haploinsufficiency in the murine integrin β3 gene (*Itgb3*) leads to a reduction in plasma-membrane levels of SERTs, which are the main target of SSRIs (Mazalouskas et al., 2015). These effects cause an increased potency of citalopram and paroxetine in the TST (Mazalouskas et al., 2015). Recapitulation of a coding polymorphism in the human *ITGB3* gene (Oliver et al., 2014) by the knock-in of Pro^32^Pro^33^ in *Itgb3* also reduces SERT serotonin reuptake, via integrin αvβ3’s actions on intracellular signaling pathways (Dohn et al., 2017). Studies in human and mouse models also have linked integrin β3 with antidepressant response (Fabbri et al., 2013; Probst-Schendzielorz et al., 2015; Rzezniczek et al., 2016; Oved et al., 2017).

In this study, we explore the role of integrin αvβ3 in modulating citalopram response in the TST. We capitalized on common signaling features observed in genetically altered *Itgb3* mice to identify novel pathways that can be targeted for antidepressant response in the future. These are the first studies examining the role of integrin αvβ3 in antidepressant response, beyond those focusing on the serotonin system.

## Materials and Methods

### Animals

Mouse studies were performed following Vanderbilt Institutional Animal Care and Use Committee guidelines under protocols M/12/167 and M/15/014. Conditional deletion of *Itgb3* was obtained by crossing floxed *Itgb3* mice (Morgan et al., 2010) with *Nestin*-Cre mice [B6.Cg-Tg (Nes-cre)1Kln/J; Jackson Lab, #003771 (Tronche et al., 1999)], which were backcrossed five generations into C57BL/6 background. Knock-in mice used in this study were generated from crosses of heterozygous C57BL/6 mice expressing one Pro^32^Pro^33^ knock-in *Itgb3* allele (Oliver et al., 2014). All other experiments were performed on C57BL/6 mice bred in house. Mice were group-housed with their littermates, maintained on a 12-h light-dark cycle, and provided with food and water *ad libitum*. We utilized mice of both sexes (8–20 weeks of age). All experimenters were blinded to the genotypes.

### Tail Suspension Test

**FIGURE 1:**
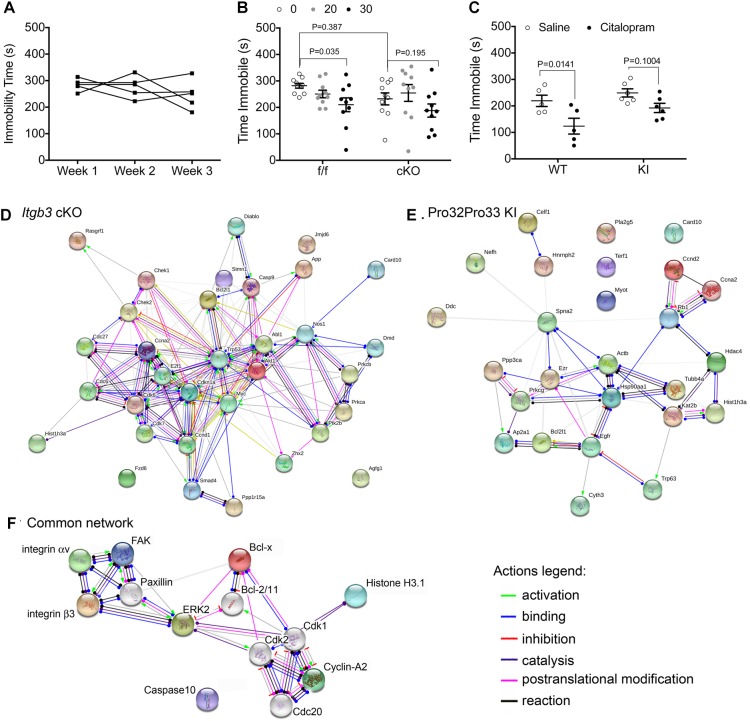
Genetic alterations in integrin αvβ3 function disrupt citalopram responses in the TST. **(A)** Effects of repeated TST testing on immobility time in C57BL/6 mice. Mice were tested after injection with saline for 6 min in the TST. Experiment was repeated once weekly, for three consecutive weeks. Repeated measures one-way ANOVA: Time *F_(1.647,6.590)_* = 1.064, *P* = 0.3825; Individual (between rows) *F_(4,8)_* = 0.7768, *P* = 0.5703. **(B)** Citalopram dose–response curve in floxed *Itgb3* lacking or expressing Cre under the control of the *Nestin* promoter (cKO). Two-way repeated measures (RM) ANOVA citalopram effect: *F_(2,36)_* = 6.172, *P* = 0.005; genotype effect: *F_(1,18)_* = 0.8719, *P* = 0.3628; interaction effect: *F_(2,36)_* = 1.057, *P* = 0.379; subject (matching): *F_(18,36)_* = 2.597, *P* = 0.0072. Bonferroni-corrected post-tests: f/f: saline vs. 30 mg/kg: *P* = 0.035, *N* = 10; cKO: saline vs. 30 mg/kg: *P* = 0.195, *N* = 10. Saline f/f vs. cKO: *P* = 0.387. **(C)** Immobility time in mice expressing Ser^32^Gln^33^ (WT) or Pro^32^Pro^33^ (KI) integrin β3 after dosing intraperitoneally (IP) with 30 mg/kg citalopram or saline control. Two-way repeated measures (RM) ANOVA citalopram effect: *F_(1,9)_* = 16.70, *P* = 0.0027; genotype effect: *F_(1,9)_* = 4.557, *P* = 0.0615; interaction effect: *F_(1,9)_* = 1.081, *P* = 0.3257; subject (matching): *F_(9,9)_* = 1.536, *P* = 0.2664. Bonferroni-corrected post-tests: WT: saline vs. 30 mg/kg: *P* = 0.0141, *N* = 5; KI: saline vs. 30 mg/kg: *P* = 0.1004, *N* = 6. **(D–F)** Schematic diagrams of protein networks identified in kinome studies. Synaptosomes were isolated from *Nestin*Cre and floxed littermates **(D)** or WT and KI littermates **(E)**, and protein extracts were analyzed using antibody microarrays. Target proteins were converted to mouse gene codes for input into the online STRING software, where network analysis was performed. Shown here are action outputs where each line linking gene nodes denotes a molecular action, as depicted in the legends. **(D)** Network linking gene products with altered expression or phosphorylation levels between *Nestin*Cre and floxed littermates. **(E)** Network of gene products that are altered in KI samples when compared to wild-type littermates. **(F)** Network of proteins that are commonly modified by genetic alterations in *Itgb3*. In this diagram, we replaced *Mus musculus* gene names by protein names for clarity. Colored nodes, including both subunits of the integrin αvβ3 receptor, FAK, and ERK2, were added during input. Nodes shown in white were added by STRING.

An automated TST device (Med Associates, St. Albans, VT, United States) was used to measure the duration of behavioral immobility. Each mouse had its tail passed through a clear 3 cm plastic tube before being suspended by the tail with tape to a vertical aluminum bar connected to a strain gauge. The following settings were used in all experiments: threshold 1: 7; gain: 8; time constant: 0.25; and resolution: 200 ms. Citalopram (R/S citalopram hydrobromide; Sigma, St. Louis, MO, United States) was prepared fresh daily by dissolving the powder in 0.9% sterile saline. Drug was administered by intraperitoneal injection in a volume of 0.01 ml/g body weight and the dose was 0, 5, 20, or 30 mg/kg, calculated as the weight of the base. Mice were injected with drug or saline 30 min before a 6 min TST. Each mouse was tested two times in the TST, with 1 week between testings, which did not significant alter immobility time (Figure 1A). A counterbalanced design was used, where half of the animals of each genotype received citalopram in 1 week and the other half in the following week. Data was analyzed by two-way repeated measures ANOVA over the 6 min period for drug vs. genotype comparisons.

A second set of experiments tested immobility responses to citalopram in the presence of kinase inhibitors (ToCris, Minneapolis, MN, United States). Three cohorts were used: two for the FAK inhibitor PF-573228 (prepared in DMSO, diluted in saline with a final concentration of 12.5% DMSO and 2.5 mM of inhibitor) and one for the MEK inhibitor SL-327 (prepared in DMSO, diluted with saline with a final DMSO concentration of 12.5% and 1.5 mM SL-327). In these cohorts, mice received saline or citalopram via intraperitoneal injection. After 10 min, kinase inhibitor or 12.5% DMSO in saline (vehicle) were administered intranasally (2.5 μl per nostril) and were then tested in the TST after 20 min. Drugs were administered intranasally as it allows the delivery of compounds that do not cross the blood–brain barrier directly into the brain (Hanson and Frey, 2008; Hanson et al., 2013). Mice were anesthetized by inhaled isoflurane at 5% and a single volume (2.5 μl/nostril) of drug or vehicle were delivered slowly dropwise to the nares using a pipetman while the mouse was in a supine position. Each mouse was randomly assigned to a combination of saline/vehicle, saline/inhibitor, citalopram/vehicle or citalopram/inhibitor for week 1 and another combination for testing on a second week. In these experiments, data was analyzed by a two-way ANOVA and group comparisons were performed using Bonferroni corrections. Detailed statistical results showing *F_(DFn,DFd)_* and *P* values for each experiment are described in the figure legends.

### Marble Burying

A novel cage was prepared with a layer of Harlan T.7089 Diamond Soft bedding (Harlan Laboratories, Indianapolis, IN, United States) covering the floor. This layer was 3 cm thick to allow burying of glass marbles of 1.5 cm diameter. Each mouse was removed from the TST apparatus and allowed to acclimate in the novel cage for 30 min. Following the acclimation period, the mouse was briefly removed from the novel cage, and 20 blue glass marbles were placed in a four-by-five grid on top of the bedding, with each marble spaced 2 cm apart. The mouse was then returned to the novel cage and given 30 additional minutes to explore and interact with the marbles without interference. After this period, the number of marbles buried was quantified. Data was analyzed using two-way ANOVA and post-tests were corrected for multiple testing using Bonferroni. Detailed statistics are presented in the figure legends.

### Isolation of Presynaptic Boutons and Antibody Array

Each array experiment consisted of two mice, one genetically modified (*Nestin*-Cre cKO or Pro^32^Pro^33^ *Itgb3* KI) and one wild-type littermate, euthanized for dissections of midbrains. A total of three biological replicates were performed for cKO mice, and two for KI mice. Each array was completed in 1 day, including synaptosomal preparation, protein labeling, and incubation with microarray slide, and imaged the following day. We utilized a commercially available microarray kit (Panorama^®^ Antibody Microarray (Cat. Number CSAA1), Sigma-Aldrich; St. Louis, MO, United States) and followed the instructions for protein extraction, labeling, hybridization, and analysis as provided by the manufacturer. Synaptosomes were prepared as described previously (Phillips et al., 2001). Detailed description of the antibody array procedures can be found in the Supplementary Material. In the first biological replicate genetically modified samples were labeled with Cy3, whereas the wild-type control was labeled with Cy5. In the following replicate, the labels were reversed to compensate potential bias of binding of Cy3 and Cy5 to the protein samples. After incubation with proteins samples, the slides were washed and scanned using an Odyssey^®^ Imaging system (LI-COR Biotechnology, Lincoln, NE, United States). Images of scanned antibody microarrays were gridded and linked to a protein print list. A blinded reviewer identified missing spots and background signal. Two levels of normalization were used: log ratios of Cy5 to Cy3 were determined between array replicates to determine whether there was a bias for the fluorophore (e.g., compare cKO Cy3 and cKO Cy5 from two experiments). Then, within each biological replicate, data was normalized to GAPDH fluorescence intensity. The Cy5 results were divided by the Cy3 results for each individual protein; proteins of interest were identified by a Cy5/Cy3 ratio higher than 2 or lower than 0.5 in all biological replicates. The list of proteins was converted into a list of *Mus musculus* genes for network analysis in STRING^1^ using the actions output.

## Results

### Genetic Alteration in *Itgb3* Prevents Citalopram From Reducing Immobility Time in the TST

To examine the role of integrin αvβ3 on the citalopram response in the TST, we generated mice lacking integrin β3 expression in neuronal and glial precursors in the brain (cKO). A dose–response curve for citalopram revealed that, while 30 mg/kg citalopram elicited decreases in immobility in floxed littermates, no reductions in immobility time were observed in cKO mice (Figure 1B). We then tested the effects of 30 mg/kg citalopram on mice expressing either Ser^32^Gln^33^ (WT) or Pro^32^Pro^33^ (KI) integrin β3, as the latter present alterations in the serotonin system (Dohn et al., 2017). No significant reductions in immobility were observed in KI mice, whereas citalopram reduced immobility times in WT controls (Figure 1C).

### Kinome Analysis of Synaptosomes Isolated From cKO and KI *Itgb3* Mice

As both genetic deletion of *Itgb3* in the brain and constitutive activation of integrin αvβ3 led to diminished sensitivity to citalopram in the TST, we hypothesized that this acute response to citalopram depends on common signaling pathways modified in both mouse lines. To identify potential signaling pathways that are commonly altered by integrin αvβ3 loss- or gain-of-function, we performed kinome analysis using antibody arrays. We utilized a commercially available antibody microarray that allows for simultaneous quantification of phosphorylated and non-phosphorylated proteins in control and target groups (Kopf et al., 2005). Pathway analysis comparing cKO and floxed *Itgb3* controls (Figure 1D) revealed enrichment in proteins involved in the regulation of cell cycle (GO: 0051726. False discovery rate *P* = 6.59^-13^: *Abl1*, *Akt1*, *App*, *Bcl2l1*, *Ccnd1*, *Ccna2*, *Cdkn1a*, *Cdc6*, *Cdc27*, *Cdk6*, *Cdk7*, *Check1*, *Check2*, *E2f1*, *Myc1*, *Prkca*, *Trp53*), some of which are also involved in intracellular signal transduction (GO: 0035556. False discovery rate: 2.09^-10^: *Abl1*, *Akt1*, *Card10*, *Ccna2*, *Casp9*, *Chek2*, *Diablo*, *Dmd*, *E2f1*, *Myc*, *Nos1*, *Prkca*, *Ptk2b*, *Rasgrf1*, and *Smad4*). The analysis of proteins altered by constitutive activation of αvβ3 revealed 25 gene products altered in KI samples, 11 of which participate in macromolecular subunit organization (Figure 1E. GO: 0043933. False discovery rate: 0.000453: *Actb*, *Ezr*, *Hdac4*, *Hist1h3a*, *Hsp90aa1*, *Kat2b*, *Tubb4a*, *Trp63*, and *Rb* form a protein complex, whereas *Terf1*, *Nefh*, and *Celf1* do not participate in a macromolecular complex).

The list of common proteins altered by both gain- and loss-of-function in integrin αvβ3 consists of: Bcl-x (encoded by *Bcl2l1*), caspase recruitment domain family member 10 (*Card10*), cyclin A2 (*Ccna2*) and histone H3.1 (*Hist1h3a*), which do not, as a group, consist of a single signaling pathway. To identify potential kinases that are proximal to integrin αvβ3 that could alter the proteins identified by the antibody array, we added *Itgav* and *Itgb3* and *Ptk2* (which encodes for FAK, the downstream focal adhesion kinase) and allowed STRING to add up to 10 nodes, generating the network shown in Figure 1F. In addition to FAK, paxillin and ERK2 (mitogen activated protein kinase 1, encoded by *Mapk1*) were necessary nodes linking the integrin αvβ3 receptor and the cell cycle proteins identified in the antibody arrays. Thus, we targeted those pathways in the behavioral experiments that followed.

### Inhibition of FAK and ERK Prevents Citalopram From Reducing Immobility Time in the TST

**FIGURE 2:**
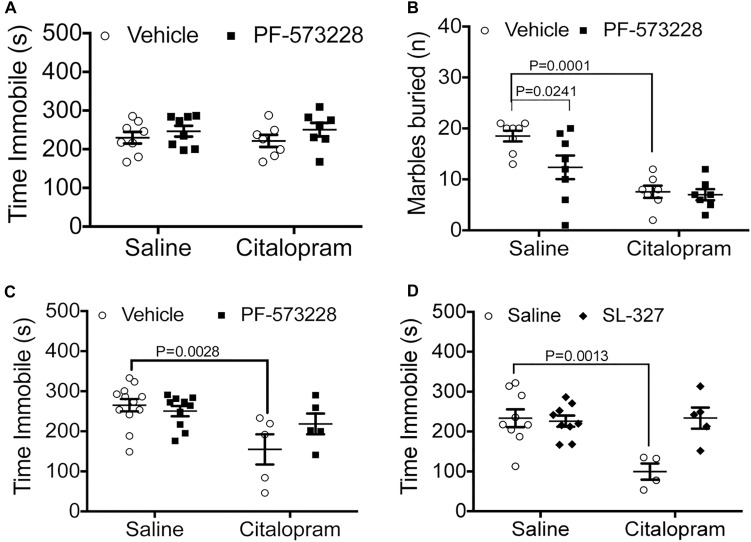
Pharmacological manipulation of FAK and ERK alter the efficacy of cirtalopram in the TST. **(A,B)** Mice were dosed IN with the FAK inhibitor PF-573228 (2.5 mM) or saline 10 min after receiving an IP injection with a sub-optimal dose of citalopram (15 mg/kg). **(A)** FAK inhibition on immobility time in the TST. Two-way ANOVA: citalopram effect: *F_(1,28)_* = 0.6715, *P* = 0.4195; PF-573228 effect: *F_(1,28)_* = 0.4201, *P* = 0.5222; interaction effect: *F_(1,28)_* = 0.1139, *P* = 0.7383. **(B)** FAK inhibition on marble burying behavior. Two-way ANOVA interaction effect: *F_(1,26)_* = 3.161, *P* = 0.0871; FAK inhibitor effect: *F_(1,26)_* = 4.595, *P* = 0.0416; Citalopram effect: *F_(1,26)_* = 27.24, *P* < 0.0001. Bonferroni-corrected post-tests: saline_IP_ + vehicle_IN_ vs. citalopram_IP_ + vehicle_IN_: *P* = 0.0001, Saline_IP_ + vehicle_IN_ vs. saline_IP_ + PF-573228_IN_: *P* = 0.0241, Saline_IP_ + vehicle_IN_ vs. citalopram + PF-573228_IN_: *P* = 0 < 0.0001. Number of mice for **(A,B)**: Saline_IP_ + vehicle_IN_ *N* = 8; citalopram_IP_ + vehicle_IN_ *N* = 7; Saline_IP_ + PF-573228 *N* = 8; Citalopram_IP_ + PF-573228_IN_ *N* = 7. **(C)** Citalopram response (30 mg/kg) in the TST was measured after intranasal (IN) administration of the FAK inhibitor PF-573228 (2.5 mM). Two-way ANOVA interaction effect: *F_(1,28)_* = 3.357, *P* = 0.0776; FAK inhibitor effect: *F_(1,28)_* = 1.300, *P* = 0.2639; citalopram effect: *F_(1,28)_* = 11.07, *P* = 0.0025. Bonferroni post-tests: Saline_IP_ + vehicle_IN_ vs. citalopram_IP_ + vehicle_IN_: *P* = 0.0028. Number of animals: saline_IP_ + vehicle_IN_ *N* = 12; citalopram_IP_ + vehicle_IN_ *N* = 5; saline_IP_ + PF-573228 *N* = 10; Citalopram_IP_ + PF-573228_IN_ *N* = 5. **(D)** Citalopram response in the TST was measured after intranasal (IN) administration of the MEK inhibitor SL-327 (1.5 mM). Two-way ANOVA interaction effect: *F_(1,23)_* = 10.06, *P* = 0.0043; MEK inhibitor effect: *F_(1,23)_* = 8.059, *P* = 0.0093; citalopram effect: *F_(1,23)_* = 7.989, *P* = 0.0096. Bonferroni-corrected post-tests: Saline_IP_ + vehicle_IN_ vs. citalopram_IP_ + vehicle_IN_: *P* = 0.0013. Number of animals: saline_IP_ + vehicle_IN_ *N* = 9; citalopram_IP_ + vehicle_IN_ *N* = 4; saline_IP_ + SL-327_IN_ *N* = 9; citalopram_IP_ + SL-327_IN_ *N* = 5.

Immediately downstream of integrin αvβ3 activation lies FAK recruitment to focal adhesions, phosphorylation, and activation, which are all necessary steps for ERK activation. Therefore, we tested whether inhibition of FAK by intranasal administration of PF-573228 (2.5 μl per nostril at 2.5 mM) could potentiate a suboptimal dose of citalopram in C57BL/6 mice. When administered intraperitoneally, neither 15 mg/kg citalopram (Crowley et al., 2005, 2006) or PF-573228 administration had effects on immobility time (Figure 2A). To examine whether these negative results were due to an inability of the drugs to reach the central nervous system, we exposed mice to the marble burying test. In this test, intranasal PF-573228 significantly decreased the number of marbles buried when compared to vehicle alone without enhancing the effect of citalopram (Figure 2B). These data suggest that FAK inhibition may have anxiolytic effects, but not antidepressant effects when tested in a behavior despair paradigm.

We then examined the potential for inhibition of FAK to influence the effective dose of citalopram (30 mg/kg). In this paradigm, we observed that intranasal PF-573228 administration prevented citalopram from reducing time immobile in the TST, recapitulating the effects observed in genetically modified *Itgb3* mice (Figure 2C).

We then examined whether downregulation of ERK1/2 alter citalopram responses by the inhibiting upstream kinase MEK1 with SL-327 (2.5 μl/nostril at 1.5 mM, or vehicle). We observed that mice dosed with citalopram had a significant reduction in immobility time, whereas those dosed with both SL-327 and citalopram had no alterations in immobility time, when compared to vehicle controls (Figure 2D). Taken together, these data indicate that inhibition of either FAK or ERK signaling pathways prevent the positive actions of citalopram in the TST.

## Discussion

Here we provide evidence that appropriate integrin αvβ3 function is necessary for citalopram response in the TST. We show that genetic alteration in the murine integrin β3 gene (*Itgb3*) and inhibition of signaling pathways downstream of integrin αvβ3 prevent citalopram from reducing immobility time in this *in vivo* model.

The genetic models utilized in this study differentially alter integrin function in the brain: the *Nestin*Cre conditional knockout line (cKO) eliminates integrin αvβ3 activity, whereas the Pro^32^Pro^33^ knock-in line (KI) has constitutively activated FAK-dependent signaling (Dohn et al., 2017). Elevated FAK phosphorylation in serotonergic synapses in the Pro^32^Pro^33^ line likely results in reduced focal adhesion turnover, which could lead to reduced neuronal motility or synapse formation/pruning that occur during development (Beggs et al., 2003; Xie et al., 2003; Rico et al., 2004; Xie and Tsai, 2004; Chacon et al., 2012; An et al., 2018). As both these lines have in common the loss of citalopram response in the TST, either dynamic activation of integrin αvβ3 signaling or integrin αvβ3-dependent circuit formation is necessary for the increased fighting response triggered by citalopram. We tested the former hypothesis by first identifying pathways that are altered in both mouse lines, followed by acute inhibition of kinases that are converging nodes in the cKO/KI signaling pathways. We exposed mice to citalopram, followed by FAK or MEK inhibitors, and observed no antidepressant response in the TST. Although the role of ERK in stress response has been examined, where ERK phosphorylation is enhanced upon exposure to the TST (Iniguez et al., 2010; Galeotti and Ghelardini, 2012; Leem et al., 2014) and ERK phosphorylation is modified with antidepressant use (Carlini et al., 2012; Licznerski and Duman, 2013), few studies have examined the involvement of ERK phosphorylation in the actions of behavior paradigms in response to antidepressants (Zeni et al., 2012). Importantly, these inhibitors had no effects on their own, indicating that FAK and ERK modulate citalopram response, but do not exert antidepressant effects by themselves. Finally, these results suggest that TST immobility is altered by integrin αvβ3 pathways in a SERT-independent fashion, and may reveal more effective targets.

The significance of these studies is thus far limited to citalopram responses in this acute measurement of behavior despair. Still, understanding the relationship between the acute effects of pharmacological treatment in the TST and the chronic effects of these drugs in the clinical setting is important to identify novel molecular targets that may be more efficacious in treatment of mood disorders. Many of the studies targeted at revealing the genetic basis for antidepressant response have yielded few results. Single-cell analysis of peripheral cells have pointed to alterations in multiple signaling pathways (Lago et al., 2018), and pharmacogenomics of antidepressant response fail to generate consistent results (Fabbri et al., 2018; Madsen et al., 2018; Rosenblat et al., 2018). Here, we propose that taking into consideration the role of serotonin in mood disorders, and utilizing a murine model with strong predictive value, can reveal molecular targets that may have higher efficacy in the clinic.

## Author Contributions

HP and RK performed experiments and analyzed data. AC and MD designed and performed experiments, analyzed data, and wrote the manuscript.

## Conflict of Interest Statement

The authors declare that the research was conducted in the absence of any commercial or financial relationships that could be construed as a potential conflict of interest.

## Footnotes

1 https://string-db.org
