# Supplementary material for: Integrin αVβ3 Function Influences Citalopram Immobility Behavior in the Tail Suspension Test
Source: Front Neurosci. 2019 Feb 6;13:70. doi: 10.3389/fnins.2019.00070 (PMC6372549; doi:10.3389/fnins.2019.00070)
Supplement: Supplementary file 1 [file Table_1.DOCX]

Supplemental Materials and Methods:

Details of the Antibody Microarrays are described below:

Mouse tissue was homogenized in 5 ml ice-cold 0.32M sucrose/4.2 mM HEPES pH 8.0 using a Teflon-glass tissue homogenizer (Wheaton Instruments, Millville, NJ). The homogenized tissue was centrifuged at 1,000*g* for 10min at 4°C and synaptosomes were collected by centrifugation at 10,000*g* for 10min at 4°C. Synaptosomal proteins were extracted using the Extraction/Labeling buffer supplied with the microarray kit, and lysates were quantified by Bradford protein assay and normalized to 1mg/ml. The soluble 10x Laemmli buffer was added for Western blot analysis to ensure purity of presynaptic fractions ([Mazalouskas *et al.*, 2015](#_ENREF_26)). Protein extracts (1ml at 1mg/ml) were added to one vial of either Cy3 or Cy5, and free dye was removed by affinity purification using Sigma Spin columns supplied with the microarray kit. The dye-to-protein molar ratio was determined following the calculations supplied with the antibody microarray kit. Only samples with a dye-to-protein molar ratio >2 were applied to the antibody microarray as recommended in the antibody microarray kit protocol. The Cy3 and Cy5 labeled sample pairs were added at equal protein concentrations (10μg/ml each) to 5ml incubation buffer for binding of labeled proteins to antibodies.

*Nestin* Cre and f/f mouse comparisons: We repeated the array experiment three times, twice where the cKO samples were tagged with Cy3 (and controls with Cy5) and once where the cKO samples were tagged with Cy5 (and controls with Cy3). After scanning all three slides, we normalized the fluorescence data to two internal controls for fluorescence (BSA), and for protein content (GAPDH). We then compared the fluorescence intensity for the Cy3 and Cy5 channels to generate a list of proteins with either altered expression or phosphorylation levels for each slide. Only proteins observed in all three array samples (32 proteins) were considered as potential targets downstream of *Itgb3,* which were collated for the generation of a gene network (Figure 1D).

KI and WT integrin β3 mice: Synaptosomes were labeled with fluorescent tags (Cy3 or Cy5) and incubated with antibody arrays. In this experiment we ran two sets of arrays and normalized the data by fluorescence intensity and GAPDH expression. Proteins that had their expression or phosphorylation levels altered in both sets were collated for network analysis (Figure 1E).

Detailed statistical analyses:

Figure 1A:

| Repeated measures ANOVA summary |  |  |  |  |  |
| --- | --- | --- | --- | --- | --- |
| Assume sphericity? | No |  |  |  |  |
| F | 1.064 |  |  |  |  |
| P value | 0.3825 |  |  |  |  |
| Geisser-Greenhouse's epsilon | 0.8237 |  |  |  |  |
| R square | 0.2101 |  |  |  |  |
|  |  |  |  |  |  |
| Was the matching effective? |  |  |  |  |  |
| F | 0.7768 |  |  |  |  |
| P value | 0.5703 |  |  |  |  |
| Is there significant matching (P < 0.05)? | No |  |  |  |  |
| R square | 0.2348 |  |  |  |  |
|  |  |  |  |  |  |
| ANOVA table | SS | DF | MS | F (DFn, DFd) | P value |
| Treatment (between columns) | 3975 | 2 | 1987 | F (1.647, 6.590) = 1.064 | P = 0.3825 |
| Individual (between rows) | 5805 | 4 | 1451 | F (4, 8) = 0.7768 | P = 0.5703 |
| Residual (random) | 14948 | 8 | 1868 |  |  |
| Total | 24728 | 14 |  |  |  |

| Number of families | 1 |  |  | |  | | |  | |  | |  | |  |
| --- | --- | --- | --- | --- | --- | --- | --- | --- | --- | --- | --- | --- | --- | --- |
| Number of comparisons per family | 2 |  |  | |  | | |  | |  | |  | |  |
| Alpha | 0.05 |  |  | |  | | |  | |  | |  | |  |
|  |  |  |  | |  | | |  | |  | |  | |  |
| Bonferroni's multiple comparisons test | Mean Diff. | 95% CI of diff. | Adjusted P Value | | |  |  |  |  |  |  |  |  |  |
|  |  |  |  | | |  |  |  |  |  |  |  |  |  |
| Week 1 vs. Week 2 | 7.258 | -81.57 to 96.09 | > 0.9999 | | |  |  |  |  |  |  |  |  |  |
| Week 1 vs. Week 3 | 37.58 | -41.50 to 116.7 | 0.3441 | | |  |  |  |  |  |  |  |  |  |
|  |  |  |  | |  | | |  | |  | |  | |  |
|  |  |  |  | |  | | |  | |  | |  | |  |
| Test details | Mean 1 | Mean 2 | SE of diff. | n1 | | n2 | | | t | | DF | |  |  |
|  |  |  |  |  | |  | | |  | |  | |  |  |
| Week 1 vs. Week 2 | 284.6 | 277.3 | 25.41 | 5 | | 5 | | | 0.2856 | | 4 | |  |  |
| Week 1 vs. Week 3 | 284.6 | 247.0 | 22.63 | 5 | | 5 | | | 1.661 | | 4 | |  |  |

| Figure 1B:  Table Analyzed | Nestin Cre Dose-response |  |  |  |  |
| --- | --- | --- | --- | --- | --- |
|  |  |  |  |  |  |
| Two-way RM ANOVA | Matching: Stacked |  |  |  |  |
| Alpha | 0.05 |  |  |  |  |
|  |  |  |  |  |  |
| Source of Variation | % of total variation | P value |  |  |  |
| Interaction | 2.126 | 0.3579 |  |  |  |
| Citalopram Dose | 12.41 | 0.0050 |  |  |  |
| Genotype | 2.277 | 0.3628 |  |  |  |
| Subjects (matching) | 47.00 | 0.0072 |  |  |  |
|  |  |  |  |  |  |
| ANOVA table | SS | DF | MS | F (DFn, DFd) | P value |
| Interaction | 7116 | 2 | 3558 | F (2, 36) = 1.057 | P = 0.3579 |
| Citalopram Dose | 41537 | 2 | 20768 | F (2, 36) = 6.172 | P = 0.0050 |
| Genotype | 7620 | 1 | 7620 | F (1, 18) = 0.8719 | P = 0.3628 |
| Subjects (matching) | 157310 | 18 | 8739 | F (18, 36) = 2.597 | P = 0.0072 |
| Residual | 121138 | 36 | 3365 |  |  |

| Number of families | 2 |  |  | |  | | |  | |  | |  | |  |
| --- | --- | --- | --- | --- | --- | --- | --- | --- | --- | --- | --- | --- | --- | --- |
| Number of comparisons per family | 2 |  |  | |  | | |  | |  | |  | |  |
| Alpha | 0.05 |  |  | |  | | |  | |  | |  | |  |
|  |  |  |  | |  | | |  | |  | |  | |  |
| Bonferroni's multiple  comparisons test | Mean Diff. | 95% CI of diff. | Adjusted P Value | | |  |  | |  | |  |  |  |  |
|  |  |  |  | | |  |  | |  | |  |  |  |  |
| f/f |  |  |  | | |  |  | |  | |  |  |  |  |
| 0 vs. 20 | 31.12 | -29.56 to 91.81 | 0.4761 | | |  |  | |  | |  |  |  |  |
| 0 vs. 30 | 71.68 | 11.00 to 132.4 | 0.0179 | | |  |  | |  | |  |  |  |  |
|  |  |  |  | | |  |  | |  | |  |  |  |  |
| cKO |  |  |  | | |  |  | |  | |  |  |  |  |
| 0 vs. 20 | -22.22 | -82.90 to 38.46 | 0.7949 | | |  |  | |  | |  |  |  |  |
| 0 vs. 30 | 44.13 | -16.55 to 104.8 | 0.1951 | | |  |  | |  | |  |  |  |  |
|  |  |  |  | |  | | |  | |  | |  | |  |
|  |  |  |  | |  | | |  | |  | |  | |  |
| Test details | Mean 1 | Mean 2 | SE of diff. | N1 | | | N2 | | t | | DF | |  |  |
|  |  |  |  |  | | |  | |  | |  | |  |  |
| f/f |  |  |  |  | | |  | |  | |  | |  |  |
| 0 vs. 20 | 281.7 | 250.6 | 25.94 | 10 | | | 10 | | 1.200 | | 36 | |  |  |
| 0 vs. 30 | 281.7 | 210.0 | 25.94 | 10 | | | 10 | | 2.763 | | 36 | |  |  |
|  |  |  |  |  | | |  | |  | |  | |  |  |
| cKO |  |  |  |  | | |  | |  | |  | |  |  |
| 0 vs. 20 | 232.2 | 254.4 | 25.94 | 10 | | | 10 | | 0.8564 | | 36 | |  |  |
| 0 vs. 30 | 232.2 | 188.1 | 25.94 | 10 | | | 10 | | 1.701 | | 36 | |  |  |

Figure 1C:

| Table Analyzed | KI-30mg/Kg citalopram |  |  |  |  |
| --- | --- | --- | --- | --- | --- |
|  |  |  |  |  |  |
| Two-way RM ANOVA | Matching: Stacked |  |  |  |  |
| Alpha | 0.05 |  |  |  |  |
|  |  |  |  |  |  |
| Source of Variation | % of total variation | P value |  |  |  |
| Interaction | 2.301 | 0.3257 |  |  |  |
| Citalopram Dose | 35.55 | 0.0027 |  |  |  |
| Genotype | 14.90 | 0.0615 |  |  |  |
| Subjects (matching) | 29.43 | 0.2664 |  |  |  |
|  |  |  |  |  |  |
| ANOVA table | SS | DF | MS | F (DFn, DFd) | P value |
| Interaction | 2050 | 1 | 2050 | F (1, 9) = 1.081 | P = 0.3257 |
| Citalopram Dose | 31673 | 1 | 31673 | F (1, 9) = 16.70 | P = 0.0027 |
| Genotype | 13278 | 1 | 13278 | F (1, 9) = 4.557 | P = 0.0615 |
| Subjects (matching) | 26222 | 9 | 2914 | F (9, 9) = 1.536 | P = 0.2664 |
| Residual | 17072 | 9 | 1897 |  |  |

| Number of families | 1 |  |  | |  | | | |  | |  | |  | |  |
| --- | --- | --- | --- | --- | --- | --- | --- | --- | --- | --- | --- | --- | --- | --- | --- |
| Number of comparisons per family | 2 |  |  | |  | | | |  | |  | |  | |  |
| Alpha | 0.05 |  |  | |  | | | |  | |  | |  | |  |
|  |  |  |  | |  | | | |  | |  | |  | |  |
| Bonferroni's multiple comparisons test | Mean Diff. | 95% CI of diff. | Adjusted P Value | | |  | |  | |  | |  |  |  |  |
|  |  |  |  | | |  | |  | |  | |  |  |  |  |
| Saline - Citalopram |  |  |  | | |  | |  | |  | |  |  |  |  |
| WT | 95.59 | 21.63 to 169.5 | 0.0141 | | |  | |  | |  | |  |  |  |  |
| KI | 56.82 | -10.70 to 124.3 | 0.1004 | | |  | |  | |  | |  |  |  |  |
|  |  |  |  | |  | | | |  | |  | |  | |  |
|  |  |  |  | |  | | | |  | |  | |  | |  |
| Test details | Mean 1 | Mean 2 | SE of diff. | N1 | | | N2 | | | t | | DF | |  |  |
|  |  |  |  |  | | |  | | |  | |  | |  |  |
| Saline - Citalopram |  |  |  |  | | |  | | |  | |  | |  |  |
| WT | 219.2 | 123.7 | 27.55 | 5 | | | 5 | | | 3.470 | | 9 | |  |  |
| KI | 249.2 | 192.4 | 25.15 | 6 | | | 6 | | | 2.259 | | 9 | |  |  |

Figure 2A:

| Table Analyzed | FAK Inhibitor |  |  |  |  |
| --- | --- | --- | --- | --- | --- |
|  |  |  |  |  |  |
| Two-way ANOVA | Ordinary |  |  |  |  |
| Alpha | 0.05 |  |  |  |  |
|  |  |  |  |  |  |
| Source of Variation | % of total variation | P value |  |  |  |
| Interaction | 0.5477 | 0.6971 |  |  |  |
| FAK inhibitor | 7.695 | 0.1522 |  |  |  |
| Citalopram | 0.06488 | 0.8933 |  |  |  |
|  |  |  |  |  |  |
| ANOVA table | SS | DF | MS | F (DFn, DFd) | P value |
| Interaction | 278.4 | 1 | 278.4 | F (1, 26) = 0.1549 | P = 0.6971 |
| FAK inhibitor | 3911 | 1 | 3911 | F (1, 26) = 2.176 | P = 0.1522 |
| Citalopram | 32.98 | 1 | 32.98 | F (1, 26) = 0.01835 | P = 0.8933 |
| Residual | 46727 | 26 | 1797 |  |  |

| Number of families | 1 |  |  | |  | |  | |  | |  | |  |
| --- | --- | --- | --- | --- | --- | --- | --- | --- | --- | --- | --- | --- | --- |
| Number of comparisons per family | 3 |  |  | |  | |  | |  | |  | |  |
| Alpha | 0.05 |  |  | |  | |  | |  | |  | |  |
|  |  |  |  | |  | |  | |  | |  | |  |
| Bonferroni's multiple comparisons test | Mean Diff. | 95% CI of diff. | Adjusted P Value | | |  |  |  | |  |  |  |  |
|  |  |  |  | | |  |  |  | |  |  |  |  |
| Vehicle:Saline vs. Vehicle:Citalopram | 8.207 | -47.94 to 64.35 | > 0.9999 | | |  |  |  | |  |  |  |  |
| Vehicle:Saline vs. PF:Saline | -16.78 | -71.02 to 37.46 | > 0.9999 | | |  |  |  | |  |  |  |  |
| Vehicle:Saline vs. PF:Citalopram | -20.79 | -76.93 to 35.36 | > 0.9999 | | |  |  |  | |  |  |  |  |
|  |  |  |  | |  | |  | |  | |  | |  |
|  |  |  |  | |  | |  | |  | |  | |  |
| Test details | Mean 1 | Mean 2 | SE of diff. | N1 | | | N2 | t | | DF | |  |  |
|  |  |  |  |  | | |  |  | |  | |  |  |
| Vehicle:Saline vs. Vehicle:Citalopram | 229.8 | 221.6 | 21.94 | 8 | | | 7 | 0.3741 | | 26 | |  |  |
| Vehicle:Saline vs. PF:Saline | 229.8 | 246.6 | 21.20 | 8 | | | 8 | 0.7917 | | 26 | |  |  |
| Vehicle:Saline vs. PF:Citalopram | 229.8 | 250.6 | 21.94 | 8 | | | 7 | 0.9474 | | 26 | |  |  |

Figure 2B:

| Table Analyzed | FAK Inhibitor-Marble Burying |  |  |  |  |
| --- | --- | --- | --- | --- | --- |
|  |  |  |  |  |  |
| Two-way ANOVA | Ordinary |  |  |  |  |
| Alpha | 0.05 |  |  |  |  |
|  |  |  |  |  |  |
| Source of Variation | % of total variation | P value |  |  |  |
| Interaction | 5.136 | 0.0871 |  |  |  |
| ERK inhibitor | 7.467 | 0.0416 |  |  |  |
| Citalopram | 44.26 | < 0.0001 |  |  |  |
|  |  |  |  |  |  |
| ANOVA table | SS | DF | MS | F (DFn, DFd) | P value |
| Interaction | 57.57 | 1 | 57.57 | F (1, 26) = 3.161 | P = 0.0871 |
| ERK inhibitor | 83.71 | 1 | 83.71 | F (1, 26) = 4.595 | P = 0.0416 |
| Citalopram | 496.2 | 1 | 496.2 | F (1, 26) = 27.24 | P < 0.0001 |
| Residual | 473.6 | 26 | 18.21 |  |  |

| Number of families | 1 |  |  | |  | | | |  | |  | |  | |  |
| --- | --- | --- | --- | --- | --- | --- | --- | --- | --- | --- | --- | --- | --- | --- | --- |
| Number of comparisons per family | 3 |  |  | |  | | | |  | |  | |  | |  |
| Alpha | 0.05 |  |  | |  | | | |  | |  | |  | |  |
|  |  |  |  | |  | | | |  | |  | |  | |  |
| Bonferroni's multiple comparisons test | Mean Diff. | 95% CI of diff. | Adjusted P Value | | |  | |  | |  | |  |  |  |  |
|  |  |  |  | | |  | |  | |  | |  |  |  |  |
| Vehicle:Saline vs. Vehicle:Citalopram | 10.93 | 5.276 to 16.58 | 0.0001 | | |  | |  | |  | |  |  |  |  |
| Vehicle:Saline vs. PF:Saline | 6.125 | 0.6643 to 11.59 | 0.0241 | | |  | |  | |  | |  |  |  |  |
| Vehicle:Saline vs. PF:Citalopram | 11.50 | 5.848 to 17.15 | < 0.0001 | | |  | |  | |  | |  |  |  |  |
|  |  |  |  | |  | | | |  | |  | |  | |  |
|  |  |  |  | |  | | | |  | |  | |  | |  |
| Test details | Mean 1 | Mean 2 | SE of diff. | N1 | | | N2 | | | t | | DF | |  |  |
|  |  |  |  |  | | |  | | |  | |  | |  |  |
| Vehicle:Saline vs. Vehicle:Citalopram | 18.50 | 7.571 | 2.209 | 8 | | | 7 | | | 4.948 | | 26 | |  |  |
| Vehicle:Saline vs. PF:Saline | 18.50 | 12.38 | 2.134 | 8 | | | 8 | | | 2.870 | | 26 | |  |  |
| Vehicle:Saline vs. PF:Citalopram | 18.50 | 7.000 | 2.209 | 8 | | | 7 | | | 5.206 | | 26 | |  |  |

Figure 2C:

| Table Analyzed | FAK Inhibitor_30mg cit. |  |  |  | |  |
| --- | --- | --- | --- | --- | --- | --- |
|  |  |  |  |  | |  |
| Two-way ANOVA | Ordinary |  |  |  | |  |
| Alpha | 0.05 |  |  |  | |  |
|  |  |  |  |  | |  |
| Source of Variation | % of total variation | P value |  | |  |  |
| Interaction | 7.824 | 0.0776 |  | |  |  |
| ERK inhibitor | 3.029 | 0.2639 |  | |  |  |
| Citalopram | 25.80 | 0.0025 |  | |  |  |
|  |  |  |  |  | |  |
| ANOVA table | SS | DF | MS | F (DFn, DFd) | | P value |
| Interaction | 10520 | 1 | 10520 | F (1, 28) = 3.357 | | P = 0.0776 |
| ERK inhibitor | 4073 | 1 | 4073 | F (1, 28) = 1.300 | | P = 0.2639 |
| Citalopram | 34688 | 1 | 34688 | F (1, 28) = 11.07 | | P = 0.0025 |
| Residual | 87734 | 28 | 3133 |  | |  |

| Number of families | 1 |  |  | |  | |  | |  | |  | |  |
| --- | --- | --- | --- | --- | --- | --- | --- | --- | --- | --- | --- | --- | --- |
| Number of comparisons per family | 3 |  |  | |  | |  | |  | |  | |  |
| Alpha | 0.05 |  |  | |  | |  | |  | |  | |  |
|  |  |  |  | |  | |  | |  | |  | |  |
| Bonferroni's multiple comparisons test | Mean Diff. | 95% CI of diff. | Adjusted P Value | | |  |  |  | |  |  |  |  |
|  |  |  |  | | |  |  |  | |  |  |  |  |
| Vehicle:Saline vs. Vehicle:Citalopram | 110.3 | 34.42 to 186.2 | 0.0028 | | |  |  |  | |  |  |  |  |
| Vehicle:Saline vs. PF:Saline | 14.80 | -46.24 to 75.83 | > 0.9999 | | |  |  |  | |  |  |  |  |
| Vehicle:Saline vs. PF:Citalopram | 46.75 | -29.12 to 122.6 | 0.3836 | | |  |  |  | |  |  |  |  |
|  |  |  |  | |  | |  | |  | |  | |  |
|  |  |  |  | |  | |  | |  | |  | |  |
| Test details | Mean 1 | Mean 2 | SE of diff. | N1 | | | N2 | t | | DF | |  |  |
|  |  |  |  |  | | |  |  | |  | |  |  |
| Vehicle:Saline vs. Vehicle:Citalopram | 265.2 | 154.9 | 29.80 | 12 | | | 5 | 3.702 | | 28 | |  |  |
| Vehicle:Saline vs. PF:Saline | 265.2 | 250.4 | 23.97 | 12 | | | 10 | 0.6173 | | 28 | |  |  |
| Vehicle:Saline vs. PF:Citalopram | 265.2 | 218.4 | 29.80 | 12 | | | 5 | 1.569 | | 28 | |  |  |

Figure 2D:

| Table Analyzed | Erk Inhibitor |  |  | |  |  |
| --- | --- | --- | --- | --- | --- | --- |
|  |  |  |  | |  |  |
| Two-way ANOVA | Ordinary |  |  | |  |  |
| Alpha | 0.05 |  |  | |  |  |
|  |  |  |  | |  |  |
| Source of Variation | % of total variation | P value |  |  |  |  |
| Interaction | 23.49 | 0.0043 |  |  |  |  |
| ERK inhibitor | 18.81 | 0.0093 |  |  |  |  |
| Citalopram | 18.65 | 0.0096 |  |  |  |  |
|  |  |  |  | |  |  |
| ANOVA table | SS | DF | MS | | F (DFn, DFd) | P value |
| Interaction | 29832 | 1 | 29832 | | F (1, 23) = 10.06 | P = 0.0043 |
| ERK inhibitor | 23895 | 1 | 23895 | | F (1, 23) = 8.059 | P = 0.0093 |
| Citalopram | 23688 | 1 | 23688 | | F (1, 23) = 7.989 | P = 0.0096 |
| Residual | 68193 | 23 | 2965 | |  |  |

| Number of families | 1 |  |  | |  | |  | |  | |  | |  |
| --- | --- | --- | --- | --- | --- | --- | --- | --- | --- | --- | --- | --- | --- |
| Number of comparisons per family | 3 |  |  | |  | |  | |  | |  | |  |
| Alpha | 0.05 |  |  | |  | |  | |  | |  | |  |
|  |  |  |  | |  | |  | |  | |  | |  |
| Bonferroni's multiple comparisons test | Mean Diff. | 95% CI of diff. | Adjusted P Value | | |  |  |  | |  |  |  |  |
|  |  |  |  | | |  |  |  | |  |  |  |  |
| Saline:Saline vs. Saline:Citalopram | 133.9 | 49.41 to 218.4 | 0.0013 | | |  |  |  | |  |  |  |  |
| Saline:Saline vs. SL-327:Saline | 7.436 | -58.84 to 73.71 | > 0.9999 | | |  |  |  | |  |  |  |  |
| Saline:Saline vs. SL-327:Citalopram | -0.2753 | -78.69 to 78.14 | > 0.9999 | | |  |  |  | |  |  |  |  |
|  |  |  |  | |  | |  | |  | |  | |  |
|  |  |  |  | |  | |  | |  | |  | |  |
| Test details | Mean 1 | Mean 2 | SE of diff. | N1 | | | N2 | t | | DF | |  |  |
|  |  |  |  |  | | |  |  | |  | |  |  |
| Saline:Saline vs. Saline:Citalopram | 233.4 | 99.46 | 32.72 | 9 | | | 4 | 4.092 | | 23 | |  |  |
| Saline:Saline vs. SL-327:Saline | 233.4 | 225.9 | 25.67 | 9 | | | 9 | 0.2897 | | 23 | |  |  |
| Saline:Saline vs. SL-327:Citalopram | 233.4 | 233.6 | 30.37 | 9 | | | 5 | 0.009066 | | 23 | |  |  |
